# Remarks on the "Living Skeleton."

**Published:** 1825-10

**Authors:** John Boyle


ART. VI.—*Remarks on the "Living Skeleton."* By JOHN BOYLE, Esq. M.R.C.S. &c.

To those unused to visit the bedside of disease and death, the living skeleton (as he is called) exhibits a most extraordinary and pitiable appearance; but, to the physician or surgeon, the most remarkable peculiarities are the great depression of the sternum, and the singular disproportion in the comparative size of the muscles.

On looking at the face, nothing particular is observable: it merely presents the aspect of one who had lived a sedentary life,—an appearance which is strengthened by high cheek-bones and a dark complexion. It is the contrast, then, between this and other parts of the body that excites curiosity, and to which my attention was drawn during the few minutes allotted to spectators.

The muscles of the neck and those which elevate the scapulæ are powerful; those of the posterior part of the pelvis, as also the fore-arms and legs, are not very small, and are particularly firm. The gastrocnemius of the right leg is of moderate size. The humoral bones are certainly smaller than natural, and exhibit but slight traces of muscle, barely sufficient to give a feeble motion to the fore-arms. The muscles of the trunk, and those of the thighs, are very indistinct; from which it will appear, whether the work of nature or of art, that certain sets of muscles, at alternate spaces only, suffer from emaciation in a great degree: as, for example, the muscles of the face, neck, and shoulder-blades, are well defined; those of the trunk and arms are particularly small; those of the fore-arms and posterior part of the pelvis are comparatively large; whilst those of the thighs, again, are weak, and those of the legs may be pronounced strong.

The sternum is concave, instead of being convex, and appears to press the thoracic viscera most at the upper part; a circumstance which, in some measure, accounts for the action of the heart and lungs being perceptible beneath their natural situation in the chest. The abdomen has a strange bag-like appearance, as might be expected, from relaxation and want of power in its muscles. The state of the pulse, tongue, and skin, as well as the appearances of the eyes, are indicative of as good health as could possibly be expected, considering the necessarily debilitated state of the system.

The "living skeleton," like any other subject which presents a deviation from the ordinary course, can serve only for the gratification of a vague curiosity, unless attention be directed to the causes of those peculiarities which render him remarkable: then only does he become a subject of rational and useful inquiry. It is with a view to such an inquiry that I offer the following suggestions; and if, as I conjecture, art shall be found to have been conjoined with nature in producing the extraordinary results which engage public attention, those results would certainly not be rendered less interesting by the inquiry.

That extrinsic measures are frequently practised upon the human frame for interested purposes, might be proved by numberless cases; but, as most medical men are more or less acquainted with these circumstances, it may be sufficient to advert to the numerous stiff joints and wasted limbs exhibited in time of war, by soldiers and sailors desirous of quitting the public service; by itinerant mendicants at all periods, in order to extort charity; and by the Indian fanatic, for the less wise purpose of *gaining cast.*

The malformation of the chest, I admit, must have been either congenital or the result of an accident during infancy: but that such irregularity of the muscles as is manifested in this extraordinary being, is a mere frolic of nature, or altogether the result of disease or accident, appears inconsistent with experience and physiological inference; although some of them may have existed, and may have afforded encouragement for the practice of such external measures as are capable of lessening the bulk and strength of the moving powers.

The right side, over the region of the liver, bears marks of the application of numerous leeches; from which it may be inferred that there was active inflammation at some past period; and which, together with the malformation of the chest, would soon establish a state favourable for the action of local pressure, and, consequently, for the diminution of parts intended to be deprived of healthy action.

It may be said, in reply to this, that if we lessen the force of the circulation in the superior part of an extremity, the inferior portion must suffer in a greater degree; but the fact is not so, as is every day proved by the result of operations for popliteal aneurism, and other cases requiring the security of large arterial trunks, whose vitality in the more distant parts is kept up by anastomosing branches. There is one more strong reason for supposing that the less perfect development of muscle upon alternate extremities mainly depends upon art,—namely, that the muscles at the extreme parts of the body are by far the most powerful; a circumstance which is by no means likely to arise from natural causes, for, as any tyro in medicine must know, in debility and approaching dissolution, it is the remote parts of the body that suffer first, from deficiency of circulation and nervous energy.

I would only add, that the almost perfect growth of all the bones of the body is presumptive proof, at least, of an equal distribution of nutrition; and, consequently, that nature performed her duty up to the period of manhood.

